# Sex Differences in Fall Frequency, Risk Factors, and Outcomes in Parkinson's Disease: A Cross‐Sectional Analysis

**DOI:** 10.1002/mdc3.70664

**Published:** 2026-05-07

**Authors:** Joaquin A. Vizcarra, Kat Hefter, David‐Erick Lafontant, Michael Tran Duong, Ashkan Ertefaie, Brian Litt, Dani S. Bassett, Andrew Siderowf, The Parkinson's Progression Markers Initiative, Kenneth Marek, Caroline Tanner, Tanya Simuni, Andrew Siderowf, Douglas Galasko, Lana Chahine, Christopher Coffey, Kalpana Merchant, Kathleen Poston, Roseanne Dobkin, Tatiana Foroud, Brit Mollenhauer, Dan Weintraub, Ethan Brown, Karl Kieburtz, Mark Frasier, Todd Sherer, Sohini Chowdhury, Roy Alcalay, Aleksandar Videnovic, Duygu Tosun‐Turgut, Werner Poewe, Susan Bressman, Jan Hammer, Raymond James, Ekemini Riley, John Seibyl, Leslie Shaw, David Standaert, Sneha Mantri, Nabila Dahodwala, Michael Schwarzschild, Connie Marras, Hubert Fernandez, Ira Shoulson, Helen Rowbotham, Paola Casalin, Claudia Trenkwalder, Todd Sherer, Sohini Chowdhury, Mark Frasier, Jamie Eberling, Katie Kopil, Alyssa O’Grady, Maggie McGuire Kuhl, Leslie Kirsch, Tawny Willson, Emily Flagg, Tanya Simuni, Bridget McMahon, Craig Stanley, Kim Fabrizio, Dixie Ecklund, Trevis Huff, Tatiana Foroud, Laura Heathers, Christopher Hobbick, Gena Antonopoulos, John Seibyl, Kathleen Poston, Christopher Coffey, Chelsea Caspell‐Garcia, Michael Brumm, Bioinformatics Core, Arthur Toga, Karen Crawford, Tatiana Foroud, Jan Hamer, Brit Mollenhauer, Doug Galasko, Kalpana Merchant, Andrew Singleton, Tatiana Foroud, Thomas Montine, Caroline Tanner, Carlie Tanner, Ethan Brown, Lana Chahine, Roseann Dobkin, Monica Korell, Charles Adler, Roy Alcalay, Amy Amara, Paolo Barone, Bastiaan Bloem, Kathrin Brockmann, Norbert Brüggemann, Lana Chahine, Kelvin Chou, Nabila Dahodwala, Alberto Espay, Stewart Factor, Hubert Fernandez, Michelle Fullard, Douglas Galasko, Penelope Hogarth, Shu‐Ching Hu, Michele Hu, Stuart Isaacson, Christine Klein, Rejko Krueger, Mark Lew, Zoltan Mari, Connie Marras, Maria Jose Martí, Nikolaus McFarland, Tiago Mestre, Brit Mollenhauer, Emile Moukheiber, Alastair Noyce, Wolfgang Oertel, Njideka Okubadejo, Sarah O’Shea, Rajesh Pahwa, Nicola Pavese, Werner Poewe, Ron Postuma, Giulietta Riboldi, Lauren Ruffrage, Javier Ruiz Martinez, David Russell, Marie H. Saint‐Hilaire, Neil Santos, Wesley Schlett, Ruth Schneider, Holly Shill, David Shprecher, Tanya Simuni, David Standaert, Leonidas Stefanis, Yen Tai, Caroline Tanner, Arjun Tarakad, Eduardo Tolosa, Aleksandar Videnovic, Susan Ainscough, Courtney Blair, Erica Botting, Isabella Chung, Kelly Clark, Ioana Croitoru, Kelly DeLano, Iris Egner, Fahrial Esha, May Eshel, Frank Ferrari, Victoria Kate Foster, Alicia Garrido, Madita. Grümmer, Bethzaida Herrera, Ella Hilt, Chloe Huntzinger, Raymond James, Farah Kausar, Christos Koros, Yara Krasowski, Dustin Le, Ying Liu, Taina M. Marques, Helen Mejia Santana, Sherri Mosovsky, Jennifer Mule, Philip Ng, Lauren O’Brien, Abiola Ogunleye, Oluwadamilola Ojo, Obi Onyinanya, Lisbeth Pennente, Romina Perrotti, Michael Pileggi, Ashwini Ramachandran, Deborah Raymond, Jamil Razzaque, Shawna Reddie, Kori Ribb, Kyle Rizer, Janelle Rodriguez, Stephanie Roman, Clarissa Sanchez, Cristina Simonet, Anisha Singh, Elisabeth Sittig, Angela Stovall, Bobbie Stubbeman, Alejandra Valenzuela, Catherine Wandell, Diana Willeke, Karen Williams, Dilinuer Wubuli

**Affiliations:** ^1^ Department of Neurology University of Pennsylvania Perelman School of Medicine Philadelphia Pennsylvania USA; ^2^ Center for Neuroengineering and Therapeutics University of Pennsylvania Philadelphia Pennsylvania USA; ^3^ Department of Bioengineering University of Pennsylvania School of Engineering and Applied Sciences Philadelphia Pennsylvania USA; ^4^ Department of Biostatistics, College of Public Health University of Iowa Iowa City Iowa USA; ^5^ Department of Radiology University of Pennsylvania Perelman School of Medicine Philadelphia Pennsylvania USA; ^6^ Department of Biostatistics, Epidemiology & Informatics University of Pennsylvania Philadelphia Pennsylvania USA

**Keywords:** fall, NSD‐ISS, Parkinson's, risk factors

## Abstract

**Background:**

Female sex is an independent fall risk factor in Parkinson's disease (PD), yet sex‐specific fall patterns remain unclear.

**Objectives:**

To compare sex‐specific fall risk and outcomes across PD, prodromal alpha‐synucleinopathy (PAS), and healthy controls (HC); estimate fall frequency across PD progression; and assess how sex modifies fall risk and outcomes.

**Methods:**

Fall outcomes were analyzed in the Parkinson's Progression Markers Initiative. Yearly rates of rare and frequent falls were estimated by time since diagnosis. PD participants were classified as never, rare, or frequent fallers. Clinical measures included motor, cognitive, behavioral, sleep, and autonomic domains. Outcomes included injuries and healthcare utilization. Regression models adjusted for age, sex, and disease duration with Benjamini‐Hochberg correction.

**Results:**

Among 3100 participants (937 PD, 1926 PAS, 237 HC; 6977 visits), PD participants had higher odds of falling than PAS (OR 1.66, 95% CI 1.46–1.87) and HC (OR 4.03, 95% CI 3.14–5.23). In PD and PAS, females had higher odds of injuries (OR 1.50, 95% CI 1.20–1.88) and fractures (OR 1.62, 95% CI 1.15–2.29), including hip (OR 2.30, 95% CI 1.09–4.91) and upper‐extremity fractures (OR 2.67, 95% CI 1.51–4.85). Within PD, falls increased with disease duration and were higher in females (7 years: 42% vs. 32%; 14 years: 88% vs. 61%) despite milder clinical profiles. Across the Neuronal Synuclein Disease‐Integrated Staging System (NSD‐ISS) stages, fall occurrence was higher in females.

**Conclusion:**

Falls increase with disease duration and NSD‐ISS stage. Female PD participants are at greater risk despite milder symptoms, supporting sex‐specific prevention strategies.

Falls are a common and important source of morbidity and mortality in people with Parkinson's disease (PwP). PwP fall twice as often as age‐matched individuals without Parkinson's disease (PD),^1^ with 60% experiencing at least one fall,^2^ and 9% transitioning annually to monthly falls.^3^ Consequences include fractures, hospitalizations, and reduced quality of life.^4^ PwP have a three‐fold increased risk of hip fracture,^5^ and post‐fracture mortality is twice than those without PD.^6^ A substantial share of the $7.1 billion USD in excess PD‐related healthcare costs in 2017 is likely due to falls.^7^

Several clinical risk factors have been identified. Prior falls are among the strongest predictors.^8^ Motor impairment, such as freezing of gait, postural instability, and higher Hoehn & Yahr (H&Y) and Movement Disorders Society Unified Parkinson's Disease Rating Scale (MDS‐UPDRS) Part III scores are also associated with fall risk.^9^ The postural instability/gait difficulty (PIGD) phenotype carries greater risk than tremor‐dominant PD.^2^ Cognitive impairment, including global decline, slowed processing speed,^10^, ^11^ and domain‐specific inattention and executive dysfunction,^12^ also contribute. Autonomic dysfunction, particularly neurogenic orthostatic hypotension (OH), has been associated with falls due to fainting.^13^, ^14^ Sex differences are an additional, underexplored dimension. Female PwP often show milder motor phenotypes than their male counterpart, but studies suggest that female sex is an independent fall risk factor in PD.^3^ Yet it remains unclear whether fall patterns differ by sex across disease duration, a critical gap for sex‐specific risk stratification and prevention.

Using the Parkinson's Progression Markers Initiative (PPMI) dataset, we aimed to: (1) compare the sex‐specific risk of falls and outcomes among PD, prodromal alpha‐synucleinopathy (PAS), and healthy controls (HC); (2) estimate sex‐specific fall frequency across PD duration and Neuronal Synuclein Disease Integrated Staging System (NSD‐ISS);^15^ and (3) evaluate sex as a modifier of fall risk and related morbidity in fall‐prone PD patients. Our goal is to identify at‐risk patients to inform integrated prevention strategies.

## Methods

Data, protocol, and materials may be requested from the Parkinson's Progression Markers Initiative (PPMI) at https://www.ppmi-info.org. This cross‐sectional study followed STROBE reporting guidelines.^16^

### Study Design

PPMI is a multicenter, international, prospective cohort study.^17^ We first assessed fall frequency and outcomes at each visit among PD, PAS, and HC participants. We then created a cross‐sectional dataset from PD visits to form adequately sized groups of unique PwP with different fall frequencies. Each PwP was included once and assigned to a single group, as detailed in the statistical analysis.

### Participants

Briefly, PwP were required to be aged 30 years or older (at diagnosis); have a H&Y score of <3; have at least two of the following: resting tremor, bradykinesia, rigidity (must have either resting tremor or bradykinesia), OR either asymmetric resting tremor or asymmetric bradykinesia; and evidence of dopaminergic deficit consistent with PD based on dopamine transporter single‐photon emission computed tomography (DAT SPECT) imaging. PAS participants had hyposmia and/or REM Sleep Behavior Disorder (RBD) with mild deficits on DAT SPECT, without pathogenic genetic variants that increase the risk of developing PD. HC had no clinically significant neurological disorder, no first‐degree relative with PD, and normal DAT SPECT.

Data sources included: 1. Curated public data cut (v.20241211) and 2. Determination of Freezing and Falls (v.20250110). We excluded participants with known monogenic PD and those without any fall data.

### Clinical Variables

PPMI participants had comprehensive evaluations at annual study visits.^18^ We analyzed demographic and clinical variables, including age, sex (assigned at birth), race, ethnicity, years of education, body mass index (kg/m2), disease duration (months from diagnosis), levodopa equivalent daily dose (LEDD),^19^ modified Schwab and England Activities of Daily Living Scale (S&E), and NSD‐ISS.^15^ We also analyzed assessments of motor, cognitive, behavioral, sleep, and autonomic measures.^14^ Motor assessments included MDS‐UPDRS, H&Y stage, and PIGD scores.^20^ Cognitive assessments included Montreal Cognitive Assessment (MoCA), Benton Judgment of Line Orientation (BJLO), Clock Drawing Test, Modified Boston Naming Test (MBNT), Hopkins Verbal Learning Test (HVLT), Symbol Digit Modalities Test (SDMT), and Trails Making Test (TMT). Behavioral assessments included Questionnaire for Impulsive‐Compulsive Disorders in Parkinson's Disease (QUIP), Geriatric Depression Scale (GDS‐15), and State–Trait Anxiety Inventory (STAI). Sleep assessments included the Epworth Sleepiness Scale (ESS) and RBD Screening Questionnaire (RBDSQ). Autonomic assessments included Scales for Outcomes in Parkinson's disease—Autonomic Dysfunction (SCOPA) and orthostatic (supine to standing) change in systolic blood pressure. No data imputation was performed for missing clinical variables.

### Determination of Falls, Injury, and Healthcare Utilization

We assessed falls using self‐reported modified items 13 and 14 of the Unified Parkinson's Disease Rating Scale (UPDRS)—Part II,^21^ introduced in PPMI in 2019 for yearly collection. Item 13 captures falls unrelated to freezing and item 14 falls related to freezing, assessed over the last week and 12 months; we used the higher score. Fall frequency was defined as “none,” “rare,” and “frequent” based on combinations reported in Table S1. We assessed fall‐related injury and healthcare utilization via self‐reported binary questionnaires about the different fall‐related injuries and healthcare utilization in the past year. We collected the following injuries: hip fracture, upper extremity fracture, skull fracture, other fracture, head injury, laceration requiring sutures, and other injuries. We collected data on the healthcare utilizations: outpatient clinic visit, emergency department visit, inpatient hospitalization, surgery, and institutionalization. No data was imputed when fall frequency was not recorded. For visits with documented falls but missing data on injury or healthcare utilization (n = 6), these outcomes were assumed not to have occurred. All other missing data were left as missing.

### Statistical Analysis

All statistical analyses were conducted using R 4.5.0 (R Foundation for Statistical Computing, Vienna, Austria).

#### Frequency of Falls and Fall‐Related Outcomes between PD, PAS, and HC

We analyzed falls, injuries, and healthcare utilization across all cohorts and years. Logistic regression models, with PD as the reference, estimated outcome likelihood adjusting for age and sex. Wald tests assessed cohort effects; when significant (ie, cohort membership was significant), odds ratios (ORs) were derived for PD versus other cohorts and females versus males. Multiple comparisons were corrected with the Benjamini‐Hochberg (BH) procedure (False discovery rate [FDR] = 0.05, *p* < 0.028, 76 tests). We then evaluated fall frequency effects on injuries and healthcare utilization in PD and PAS cohorts using logistic models with age, sex, and cohort as covariates. BH correction was again applied (FDR = 0.05, *p* < 0.024, 64 tests). ORs were calculated for PD/PAS, frequent/rare fallers, and female/male participants.

#### Frequency of Falls and Fall‐Related Outcomes across Disease Duration in PD

In the PD cohort, fall frequency was assessed yearly and categorized by disease duration (rounded to the nearest year) and NSD‐ISS stage. We calculated the proportion of visits with falls, injuries, and healthcare utilization for each duration year and stage, and stratified this by sex. PwP could contribute data to multiple years if fall data were available. We only displayed results with more than 10 observations per disease duration year and NSD‐ISS; NSD‐ISS stage 6 did not meet this threshold.

#### Correlates of Falling between Faller Groups and Sex in PD

Because the fall questionnaire was introduced in 2019, earlier enrollees often lacked fall data in the initial years, creating incomplete longitudinal records. To reduce confounding from unmodeled repeated measures and improve reliability of classification, we required at least two consecutive years of fall data for sampling. This approach limited bias from single‐year variability or reporting error, while ensuring consistent group assignment across participants. Each individual contributed a single observation and was classified into one of three mutually exclusive groups: never, rare, or frequent fallers. Participants’ first occurrence of either “rare‐frequent” or “frequent‐frequent” fall years were labeled as “frequent fallers,” and available variables from the last observation in that timeframe were taken. These individuals were then removed from the pool of participants who could be considered as either “never fallers” or “rare fallers.” Then, participants’ first occurrence of the combination of either “none‐rare” or “rare‐rare” fall years were labeled as “rare fallers,” available variables from the last observation in that timeframe were taken, and those participants were removed from the pool of participants who could be considered as “never fallers.” Finally, participants’ first occurrence of two consecutive “none” years were sampled and labeled as “never fallers,” and available variables from the last observation in that timeframe were taken. Based on this process, an individual subject could only be counted in one category, even if they progressed from being a never faller to a frequent faller over the subsequent study visits. Due to low event counts, injuries or healthcare use were recorded if reported in either of the two sampled years.

We then compared fall groups and sexes. We summarized variables using counts and proportions, as well as means and standard deviations, as appropriate. For categorical and continuous variables, we used logistic models and linear regression models, respectively, with age, sex, and years since diagnosis as covariates. For pairwise comparisons between fall frequency groups, the reference group was set to “rare fallers.” We used BH correction for multiple comparisons (FDR = 0.05, *p* < 0.016, 213 tests).

## Results

Of 3805 individuals screened (1442 PD, 2045 PAS, 318 HC), 360 monogenic PD and 705 without fall data were excluded, yielding 3100 participants (937 PD, 1926 PAS, 237 HC) across 6977 visits. Mean age was similar across groups (PD 66.8 ± 9.4; PAS 68.1 ± 6.5; HC 67.9 ± 11.5 years). Males were most prevalent in PD (66.2%), followed by HC (61.0%) and PAS (48.8%). Falls were reported in 1607 visits. In PD, 514 visits had rare falls, and 163 visits had frequent falls (21.7% and 6.9%, respectively); in PAS, 806 visits had rare falls, and 44 visits had frequent falls (20.9% and 1.1%, respectively); and in HC, 78 visits had rare falls, and two visits had frequent falls (10.3% and 0.3%, respectively).

### Comparison of Risk of Falls, Injury, and Healthcare Utilization between PD, PAS, and HC

Across 3100 participants and 6977 visits, PwP were more likely than PAS and HC to report falls, injuries, and healthcare utilization (Table S2). Adjusted odds of falling were higher versus PAS (OR 1.66, 95% CI 1.46–1.87) and HC (OR 4.03, 95% CI 3.14–5.23). Odds of injury were 1.70 versus PAS (95% CI 1.42–2.04) and 3.26 versus HC (95% CI 2.27–4.85), and odds of healthcare utilization were 1.71 (95% CI 1.39–2.09) and 3.81 (95% CI 2.48–6.12), respectively. After adjustment for fall frequency, cohort membership no longer predicted injury or healthcare use (Table S3), whereas fall frequency remained independently associated (Table S4).

Female participants with PD and PAS were more likely than males to experience fall‐related injuries and healthcare utilization (Table S4). Adjusted analyses showed increased odds of any injury (OR 1.50, 95% CI 1.20–1.88), including any fracture (OR 1.62, 95% CI 1.15–2.29), hip fracture (OR 2.30, 95% CI 1.09–4.91), and upper extremity fracture (OR 2.67, 95% CI 1.51–4.85). Females also had higher odds of any healthcare utilization (OR 1.48, 95% CI 1.16–1.89), outpatient visits (OR 1.41, 95% CI 1.06–1.89), and emergency department visits (OR 1.49, 95% CI 1.11–1.99). No sex differences were observed for hospitalization, surgery, or institutionalization.

### Falls, Injury, and Healthcare Utilization in PD across Years since Diagnosis and NSD‐ISS Stages

**Figure 1:**
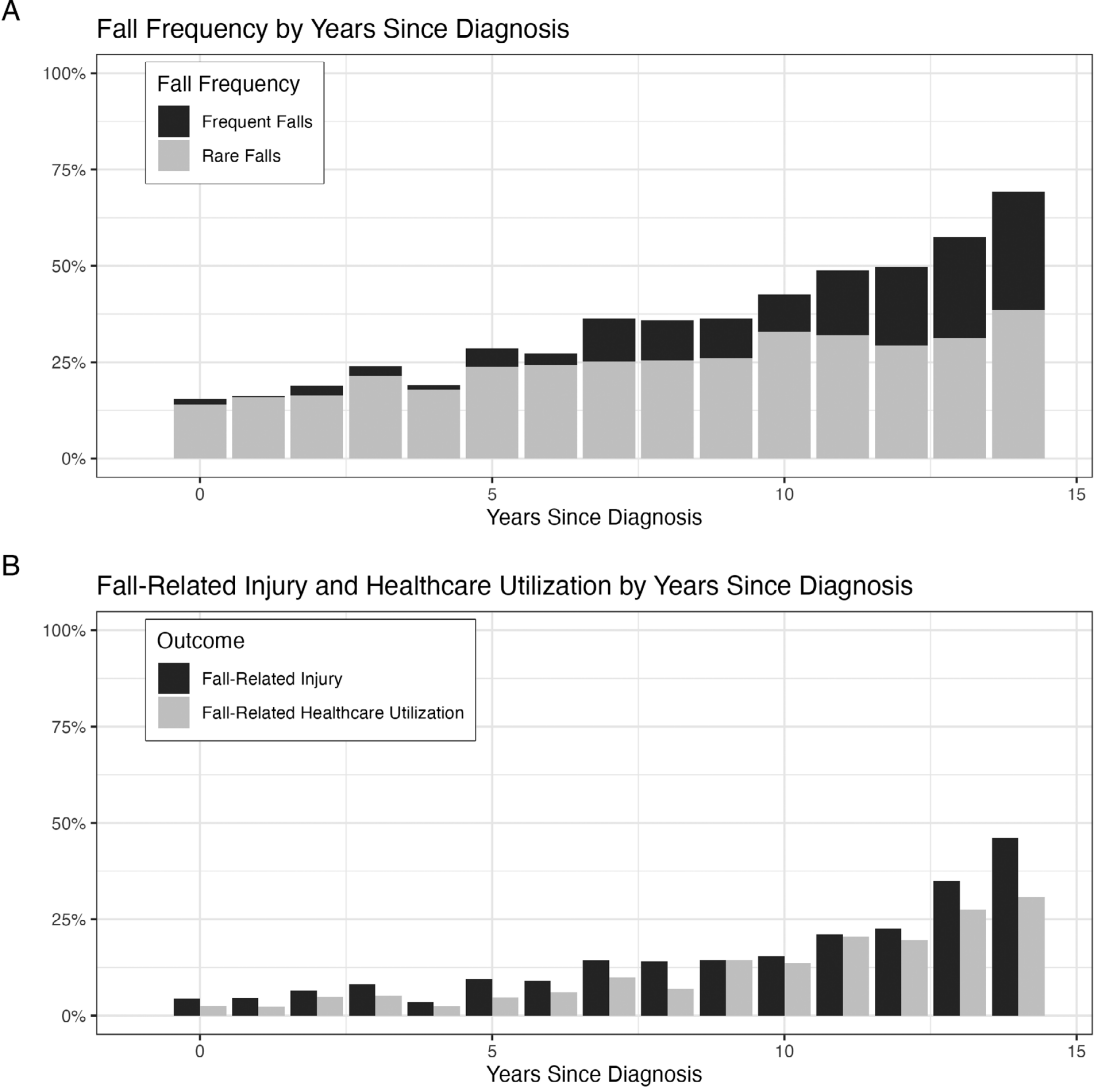
Fall frequency and outcomes across years since diagnosis in PD. In 937 participants with PD across 2372 yearly visits, the percentage of visits with (A) rare or frequent yearly falls and (B) fall‐related injury and healthcare utilization is displayed across years since diagnosis.

**Figure 2:**
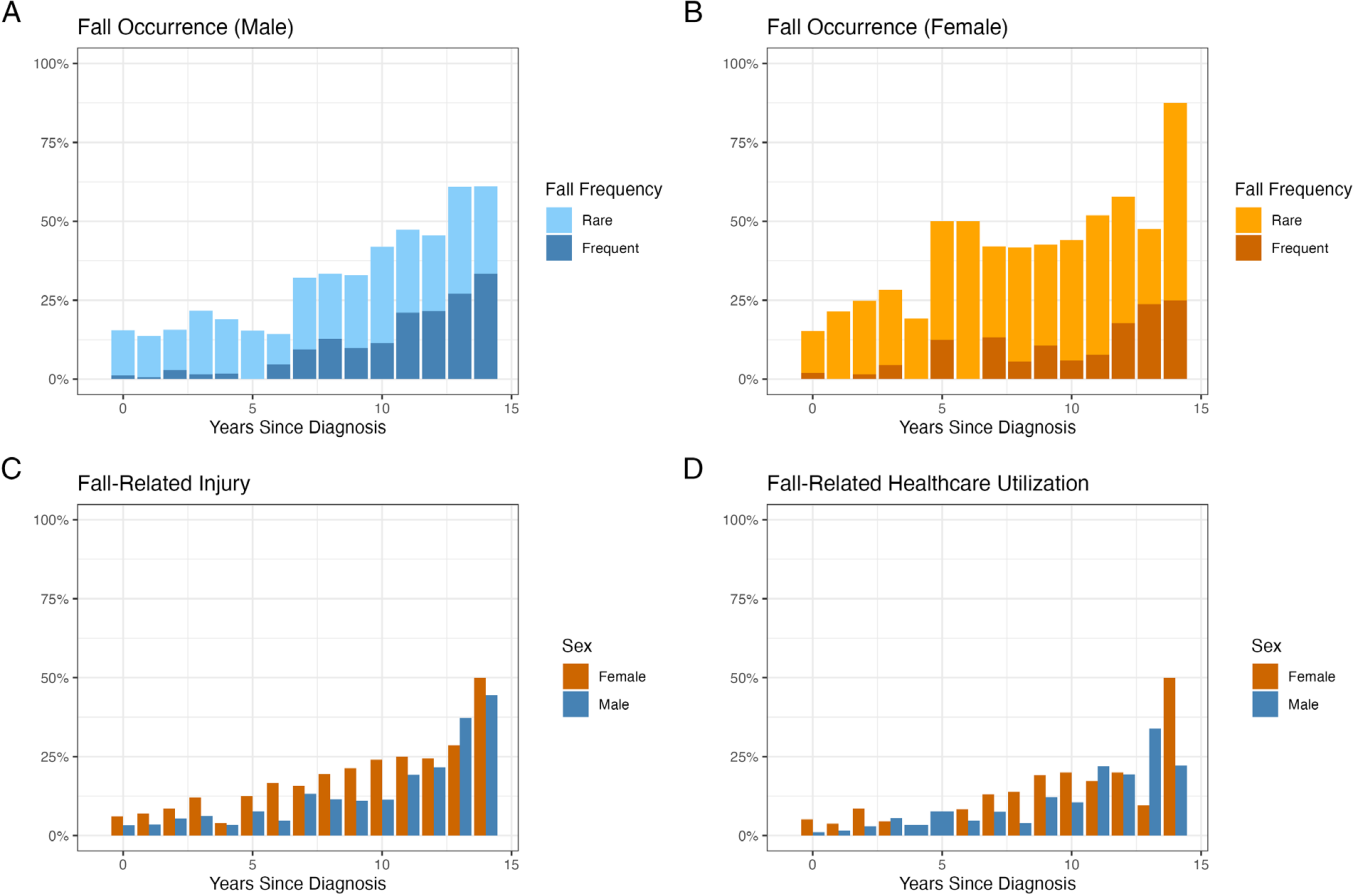
Sex differences in fall frequency and outcomes across years since diagnosis in PD. In 937 participants with PD across 2372 yearly visits, the percentage of visits with rare and frequent falls by (A) male and (B) female, (C) fall‐related injury, and (D) fall‐related healthcare utilization is displayed across years since diagnosis.

**Figure 3:**
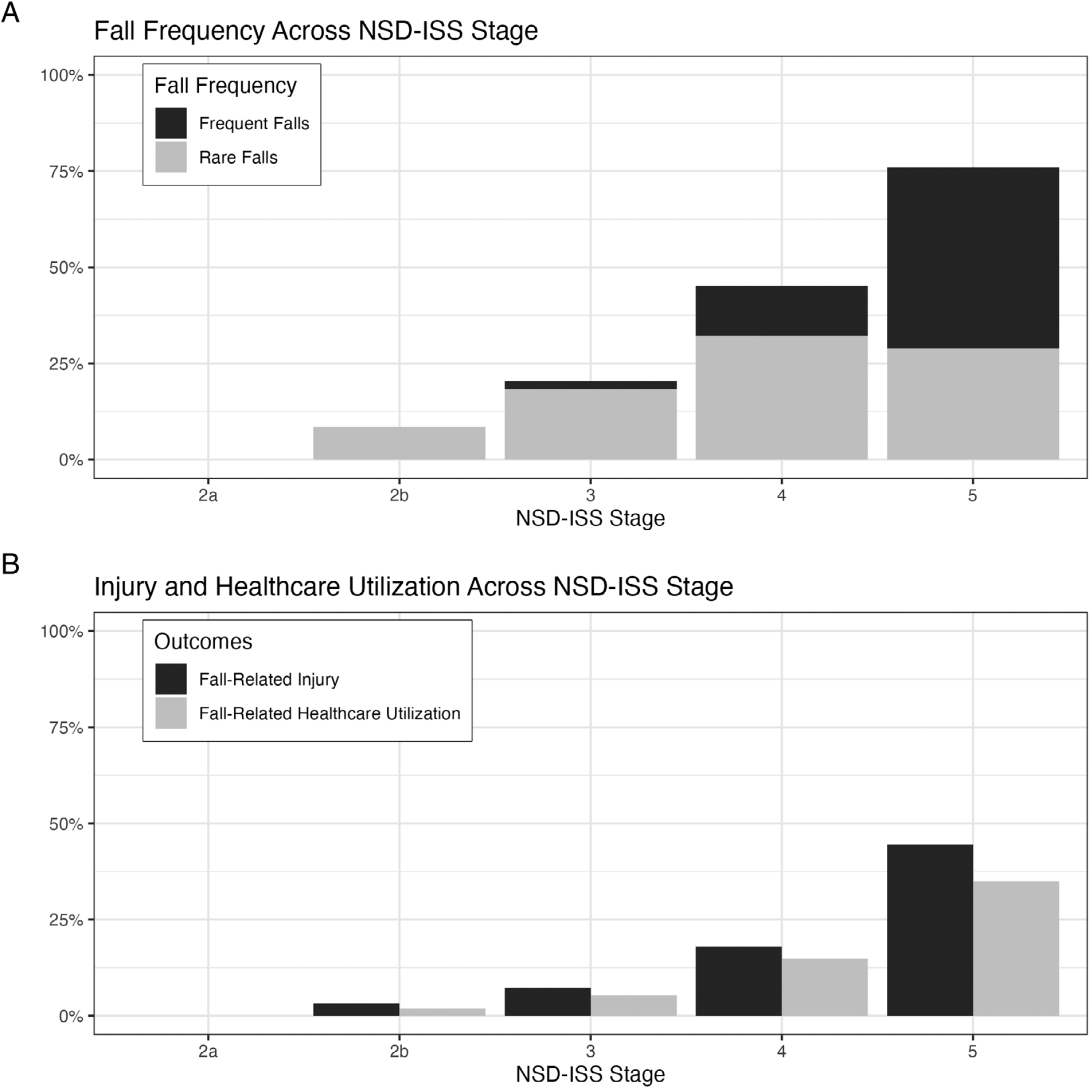
Fall frequency and outcomes across NSD‐ISS in PD. Of the 2372 visits, 2185 had NSD‐ISS data. A total of 150 visits were of participants labeled not NSD‐ISS (ie, Parkinsonism not from biologically defined neuronal synuclein disease), resulting in 2035 visits with NSD‐ISS. The percentage of visits with (A) rare or frequent yearly falls and (B) fall‐related injury and healthcare utilization is displayed across NSD‐ISS stages. NSD‐ISS, neuronal synuclein disease**‐**integrated staging system.

**Figure 4:**
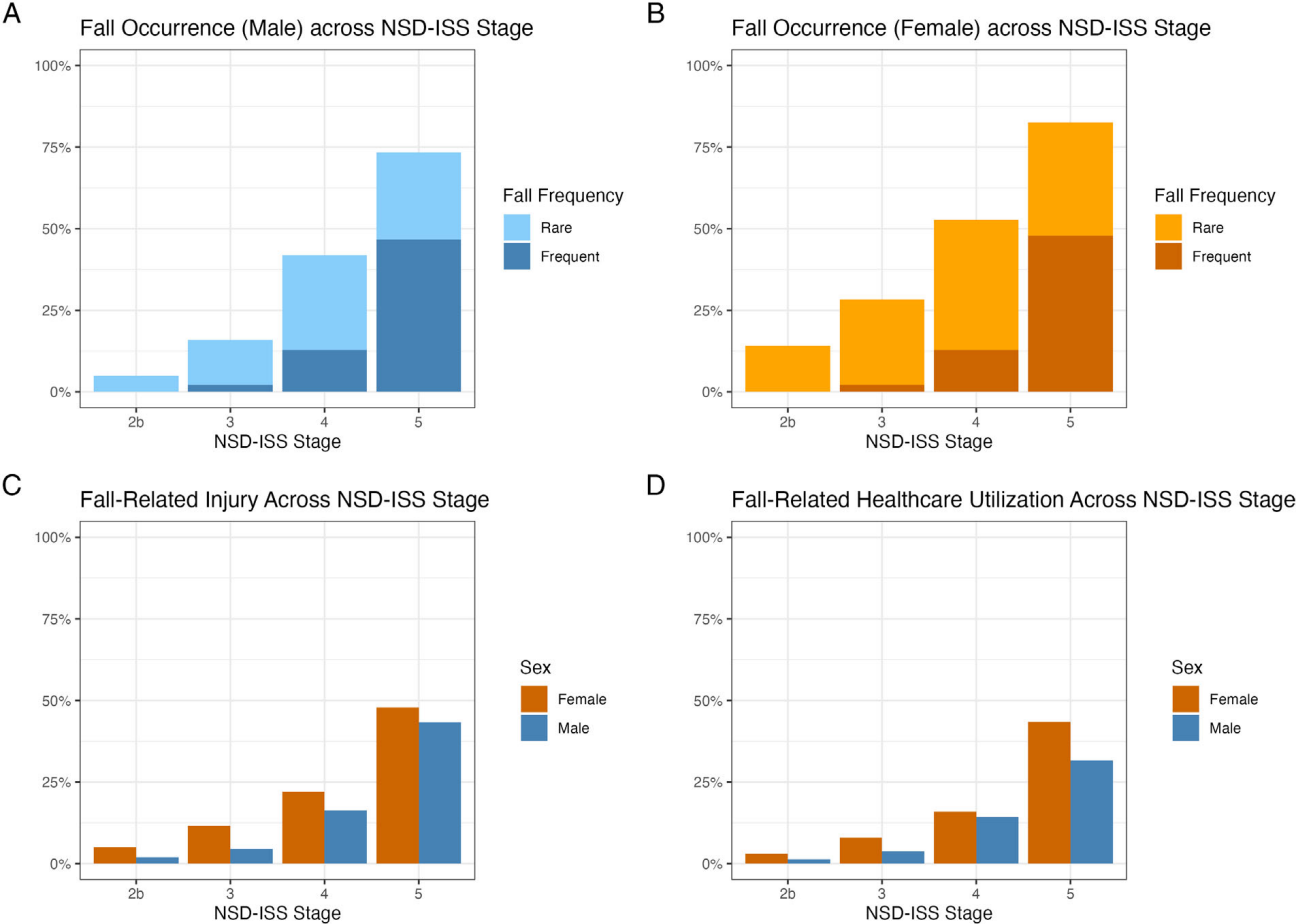
Sex differences in fall frequency and outcomes across NSD‐ISS in PD. Of the 2372 visits, 2185 had NSD‐ISS data. A total of 150 visits were of participants labeled not NSD‐ISS (ie, Parkinsonism not from biologically defined neuronal synuclein disease), resulting in 2035 visits with NSD‐ISS. The percentage of visits with rare and frequent falls by (A) male and (B) female, (C) fall‐related injury, and (D) fall‐related healthcare utilization is displayed across NSD‐ISS stages. NSD‐ISS, Neuronal Synuclein Disease**‐**Integrated Staging System.

In 937 PD participants (2372 visits), fall frequency increased with disease duration and NSD‐ISS stage. Falls occurred in 15.5%, 36.3%, and 69.2% of visits at 0, 7, and 14 years since diagnosis, respectively (Fig. 1). Among visits with falls, the percentage with a fall‐related injury increased with years since diagnosis, reaching 27.9%, 39.4%, and 66.7% at years 0, 7, and 14, respectively. Across years since diagnosis, female PwP had higher rates of falls, injuries, and fall‐related healthcare use (Fig. 2). At 14 years since diagnosis, falls were reported in 87.5% of females versus 61.1% of males, with corresponding differences in injury (50.0% vs 44.4%) and healthcare utilization (50.0% vs 22.2%). NSD‐ISS data were available for 92.1% of visits (Table S5). Falls were absent in stage 2A and limited to rare falls in stage 2B, but increased progressively across stages (Fig. 3). Frequent falls occurred in 2.1%, 12.8%, and 47.0% of visits at stages 3, 4, and 5, respectively, with similar stage‐related increases in injury (7.1%, 18.0%, and 44.6%) and healthcare utilization (5.4%, 14.8%, and 34.9%). Across stages, females consistently had higher fall occurrence, injuries, and healthcare use than males (Fig. 4), with widening differences in later stages.

### Cross‐Sectional Differences between Never, Rare, and Frequent Fallers in Unique Participants with PD

Of 937 PD participants, 605 were included and 332 excluded, primarily due to missing consecutive yearly fall data (296/332). Groups were otherwise similar, although excluded participants had shorter disease duration (2.4 vs 2.9 years, *p* = 0.02). Among unique PD participants, 371 were classified as never, 178 as rare, and 56 as frequent fallers (Fig. S1). Female PwP were more likely than males to report any fall (46.1% vs 34.9%), driven by rare but not frequent falls. Frequent fallers had longer disease duration, higher NSD‐ISS stages, and lower S&E scores. Logistic regression confirmed fall frequency correlated with NSD‐ISS stage, with frequent fallers overrepresented in later stages. LEDD did not differ significantly across groups.

#### Motor and Non‐motor Differences between Never, Rare, and Frequent Fallers and Sexes

Frequent fallers exhibited more severe motor impairment (Table 1), including higher H&Y stages, PIGD scores (ON and OFF), and MDS‐UPDRS total and Part II–IV scores (Fig. S2). Differences between never and rare fallers were minimal and largely limited to PIGD measures.

**TABLE 1:** Demographic, motor, and non‐motor differences between never, rare, and frequent fallers in the cross‐sectional PD cohort

	Never fallers (n = 371)	Rare fallers (n = 178)	Frequent fallers (n = 56)	p(Never ≠Rare)	p(Rare ≠Frequent)
Demographics					
Sex: Male	261 (70.4%)*	97 (55.5%)	43 (76.8%)*	<0.001	0.01
Age (y)	62.5 (9.4)*	62.9 (9.8)	63.5 (8.7)	0.003	0.028
Years since diagnosis	3.1 (3.0)*	6.5 (4.5)	10.1 (3.6)*	<m.p.	<0.001
Race: White	344 (93.5%)	167 (95.4%)	54 (96.4%)	0.71	0.87
Ethnicity: Not Hispanic or Latino	354 (96.6%)*	166 (92.3%)	55 (98.2%)	0.005	0.45
Education years	16 (2.8)	16 (2.9)	16.1 (2.4)	0.51	0.82
BMI	27.2 (25.3)	25.3 (4.1)	26.6 (4.4)	0.23	0.47
LEDD (mg)	294 (384)	587 (572)	829 (524)	0.083	0.54
S&E	92.5 (7)	86.8 (11.5)	70.3 (17.3)*	0.032	<m.p.
NSD‐ISS	321	153	49		
≤ Stage 3	264 (82.2%)*	90 (58.8%)	10 (20.4%)*	0.002	0.002
> Stage 3	57 (17.8%)*	63 (41.2%)	39 (79.6%)*	0.002	0.002
Not NSD‐ISS	22	13	7		
Motor Variables					
H&Y	1.8 (0.5)	2.0 (0.6)	2.6 (1.1)*	0.36	<0.001
H&Y ON	1.8 (0.5)	2.0 (0.5)	2.8 (0.9)*	0.14	<0.001
PIGD	0.3 (0.3)*	0.6 (0.5)	1.3 (0.8)*	<0.001	<m.p.
PIGD ON	0.3 (0.2)*	0.5 (0.4)	1.3 (0.8)*	<0.001	<m.p.
MDS‐UPDRS	40.0 (17.5)*	53.3 (20.9)	76.1 (22)*	<0.001	<0.001
MDS‐UPDRS ON	36.1 (16.1)*	45.9 (19.4)	69.3 (22)*	<0.001	<0.001
Part 1	6.55 (4.78)*	9.26 (5.3)	15.0 (6.8)*	<0.001	<0.001
Part 2	7.18 (5.33)*	10.6 (6.3)	20.2 (8.2)*	<0.001	<m.p.
Part 3	26.3 (11.6)*	34.5 (15.2)	43.1 (13.5)	0.001	0.12
Part 3 ON	22.4 (11.0)*	26.5 (13.3)	35.8 (12.9)*	0.016	<0.001
Part 4	1.37 (2.6)	2.89 (3.6)	5.11 (4.7)*	0.24	0.006
Cognitive variables					
MoCA	27.3 (2.4)	27.0 (3.1)	25.4 (4.2)*	0.97	0.01
BJLO	12.6 (2.4)*	11.8 (3.0)	11.8 (2.7)	0.001	0.33
SDMT	42.9 (10.4)*	38.4 (12.9)	30.8 (10.9)	0.01	0.02
TMT‐Part B	90.2 (55.2)	116 (75.6)	170 (84.8)*	0.039	0.002
Behavioral variables					
GDS‐15	1.88 (2.02)*	2.70 (2.6)	4.77 (3.5)*	<0.001	<0.001
STAI	59.8 (16.1)*	64.8 (18.5)	74.4 (21.7)*	0.005	0.001
Autonomic variables					
SCOPA	11.0 (6.9)	13.8 (6.9)	19.1 (7.9)*	0.019	<0.001
OH	55 (14.9%)	35 (20.2%)	14 (27.4%)	0.76	0.98

*Note*: Values are *n* or mean (with corresponding % or SD), as appropriate, *: statistically significant from rare, *p* < 0.016 (BH threshold for FDR 0.05). m.p. = machine precision. The following variables were not significant between faller groups and are not shown: Clock Drawing Test, Lexical Fluency Letter Test, Hopkins Verbal Learning Test, Letter Number Sequencing Test, Modified Boston Naming Test, Trail Making Test‐Part A, Questionnaire for Impulsive‐Compulsive Disorders in Parkinson's Disease, Epworth Sleepiness Scale, REM Sleep Behavior Disorder Screening Questionnaire. ON denotes the ON medication state.

Abbreviations: BJLO, Benton Judgment of line orientation; BMI, body mass index; GDS‐15, geriatric depression scale; H&Y, Hoehn and Yahr; LEDD, levodopa equivalent daily dose; MBNT, modified Boston naming test; MDS‐UPDRS, movement disorder society unified Parkinson's disease rating scale; MoCA, Montreal cognitive assessment; NSD‐ISS, Neuronal Synuclein Disease‐Integrated Staging System; OH, orthostatic hypotension; PIGD, Postural Instability and Gait Disorder; S&E, Modified Schwab and England Activities of Daily Living Scale; SCOPA, Scales for Outcomes in Parkinson's Disease–Autonomic; SDMT, Symbol Digit Modalities Test; STAI, State–Trait Anxiety Inventory; TMT, Trail Making Test.

Non‐motor burden was also greater in frequent fallers (Fig. S3), including higher MDS‐UPDRS Part I scores, depressive and anxiety symptoms, autonomic dysfunction, and deficits in processing speed, executive function, and visuospatial judgment. Although OH was more common in frequent fallers, this association was not statistically significant after adjusting for confounders.

Female PwP had milder PD symptoms, scoring five points lower on the total MDS‐UPDRS ON exam, and specifically 1.5 points lower on MDS‐UPDRS Part II, than their male counterparts (Table 2). Notably, MDS‐UPDRS Part III, PIGD, and H&Y were not significantly different between sexes on either ON or OFF states. Cognitively, female PwP outperformed males on several domains, including verbal learning and memory (eg, HVLT), verbal fluency (eg, LFLT), processing speed (eg, SDMT), and cognitive flexibility and visual attention (eg, TMT Parts A and B; Fig. S4). In contrast, male PwP performed better on visuospatial judgment (eg, BJLO). Female PwP were less likely to have sleep disturbances, with a lower percentage of RBDSQ ≥5 rates and a lower average ESS. Finally, in autonomic symptoms, female PwP were less likely to have sexual and urinary dysautonomia, and more likely to have thermoregulatory dysautonomia.

**TABLE 2:** Demographic, motor, and non‐motor sex‐based differences in the cross‐sectional PD cohort

	Male (n = 401)	Female (n = 204)	p(Male ≠Female)
Demographics			
Age (y)	66.6 (9.5)	66.8 (9.1)	0.98
Years since diagnosis	4.7 (4.3)	4.6 (4.1)	0.30
Race: White	377 (95%)	188 (93.1%)	0.30
Ethnicity: Not Hispanic or Latino	380 (95.7%)	195 (96.1%)	0.58
Education years	16.2 (2.7)	15.8 (2.8)	0.08
Fall Occurrence	140 (34.9%)	94 (46.1%)*	0.002
Rare Faller	97 (24.2%)	81 (39.7%)*	<0.001
Frequent Faller	43 (10.7%)	13 (6.4%)	0.14
BMI	27.35 (11.92)	25.14 (4.69)	0.023
LEDD (mg)	464.68 (545.22)	360.65 (367.34)*	0.001
S&E	88.28 (12.36)	89.80 (10.25)	0.14
NSD‐ISS	343	180	
≤ Stage 3	234 (68.2%)	130 (72.2%)	0.20
> Stage 3	109 (31.8%)	50 (27.8%)	
Not NSD‐ISS	29	13	
Motor variables			
H&Y	1.91 (0.59)	1.92 (0.63)	0.75
H&Y ON	1.91 (0.61)	1.88 (0.61)	0.76
PIGD	0.44 (0.50)	0.42 (0.41)	0.98
PIGD ON	0.42 (0.62)	0.39 (0.38)	0.88
MDS‐UPDRS	46.80 (21.89)	44.62 (19.64)	0.13
MDS‐UPDRS ON	42.66 (20.41)	38.63 (17.98)*	0.016
Part 1	8.21 (5.72)	7.98 (5.72)	0.65
Part 2	9.94 (7.35)	8.31 (6.28)*	0.003
Part 3	30.00 (14.26)	29.26 (12.80)	0.36
Part 3 ON	25.30 (12.57)	23.18 (11.84)	0.05
Part 4	2.29 (3.56)	2.45 (3.36)	0.70
Cognitive and behavioral variables			
BJLO	12.51 (2.55)	11.76 (2.64)*	0.004
LFLT	42.07 (13.95)	45.27 (15.07)*	0.016
SDMT	39.47 (12.04)	42.83 (10.82)*	<0.001
TMT‐Part A	44.91 (25.97)	39.14 (19.18)*	0.002
TMT‐Part B	110.09 (71.95)	93.42 (59.36)*	0.001
Sleep variables			
ESS	6.78 (4.17)	5.29 (3.90)*	<0.001
RBDSQ ≥5	186 (46.4%)	71 (35.0%)*	0.007

*Note*: Values are *n* or mean (with corresponding % or SD), as appropriate, *: statistically significant difference between male and female, *p* < 0.016 (BH threshold for FDR 0.05). m.p. = machine precision. The following variables were not significant between sexes and are not shown: Montreal Cognitive Assessment, Clock Drawing Test, Lexical Fluency Letter Test, Modified Boston Naming Test, Letter Number Sequencing Test, Questionnaire for Impulsive‐Compulsive Disorders in Parkinson's Disease, Geriatric Depression Scale, State–Trait Anxiety Inventory, orthostatic hypotension. ON denotes the ON medication state.

Abbreviations: BJLO, Benton judgment of line orientation; BMI, body mass index; ESS, Epworth sleepiness scale; H&Y, Hoehn and Yahr; LEDD, levodopa equivalent daily dose; LFLT, lexical fluency letter test; MDS‐UPDRS, movement disorder society unified Parkinson's disease rating scale; NSD‐ISS, Neuronal Synuclein Disease–Integrated Staging System; PIGD, postural instability and gait disorder; RBDSQ, REM sleep behavior disorder screening questionnaire (values ≥5 are considered positive for REM Sleep Behavior Disorder); S&E, modified Schwab and England activities of daily living scale; SDMT, symbol digit modalities test; TMT, trail making test.

#### Fall‐Related Outcome Differences between Rare and Frequent Fallers and Sexes

Frequent fallers were more likely than rare fallers to report injury (66.1% vs. 39.9%), head injury, laceration, multiple injuries, and emergency department visits (Table 3; Fig. S5). Female PwP were more likely to report any fracture (10.3% vs. 4.24%), particularly upper extremity fractures (5.4% vs. 1.8%) than their male counterparts (Table 4). Differences in hip fractures were not significant (Fig. S4). There were no differences in fall‐related healthcare utilization between sexes.

**TABLE 3:** Outcome differences between rare and frequent fallers in the cross‐sectional PD cohort

	Rare fallers (n = 178)	Frequent fallers (n = 56)	p(Rare ≠Frequent)
Injury			
Any injury	71 (39.9%)	37 (66.1%)	0.012*
Any fracture	24 (13.5%)	14 (25.0%)	0.25
Hip fracture	7 (3.9%)	3 (5.4%)	0.82
Upper extremity fracture	10 (5.6%)	8 (14.3%)	0.075
Skull fracture	2 (1.1%)	0 (0%)	1.0
Other fracture	11 (6.2%)	7 (12.5%)	0.36
Head injury	21 (11.8%)	19 (33.9%)	0.016*
Laceration	12 (6.7%)	14 (25.0%)	0.019
Other injury	34 (19.1%)	21 (37.5%)	0.005*
Multiple injuries	15 (8.4%)	19 (33.9%)	<0.001*
Healthcare utilization			
Any healthcare use	55 (30.9%)	34 (60.7%)	0.023
Outpatient visit	32 (18.0%)	24 (42.9%)	0.033
ED visit	36 (20.2%)	28 (50.0%)	0.005*
Hospitalization	11 (6.2%)	9 (16.1%)	0.33
Surgery	6 (3.4%)	7 (12.5%)	0.095
Institutionalization	2 (1.1%)	0 (0%)	1.0

ED, emergency department; Values are means with corresponding %, *: statistically significant from rare, *p* < 0.016 (BH threshold for FDR 0.05). m.p. = machine precision.

**TABLE 4:** Sex‐based fall‐related outcome differences in the cross‐sectional PD cohort

	Male (n = 401)	Female (n = 204)	p(Male ≠Female)
Injury			
Any injury	62 (15.5%)	46 (22.5%)	0.13
Any fracture	17 (4.2%)	21 (10.3%)	0.004*
Hip fracture	3 (0.7%)	7 (3.4%)	0.02
Upper extremity fracture	7 (1.7%)	11 (5.4%)	0.014*
Skull fracture	1 (0.2%)	1 (0.5%)	0.81
Other fracture	10 (2.5%)	8 (3.9%)	0.39
Head injury	28 (7.0%)	12 (5.9%)	0.58
Laceration	20 (5.0%)	6 (2.9%)	0.30
Other injury	28 (7.0%)	27 (13.2%)	0.03
Multiple injuries	21 (5.2%)	13 (6.4%)	0.32
Healthcare utilization			
Any healthcare use	51 (12.7%)	38 (18.6%)	0.06
Outpatient visit	33 (8.2%)	23 (11.3%)	0.22
ED visit	37 (9.2%)	27 (13.2%)	0.09
Hospitalization	13 (3.2%)	7 (3.4%)	0.74
Surgery	6 (1.5%)	7 (3.4%)	0.08
Institutionalization	2 (0.5%)	0 (0%)	1.0

*Note*: Values are means with corresponding %, *: statistically significant from rare, *p* < 0.016 (BH threshold for FDR 0.05). m.p. = machine precision.

Abbreviation: ED, emergency department.

## Discussion

In this large, multicohort study, we found that falls were significantly more common in PwP, with odds about 1.5‐fold higher than PAS and over fourfold higher than HC. Fall‐related injuries and healthcare utilization were also more frequent, largely explained by increased fall frequency and increasing with years since diagnosis and NSD‐ISS stage. Across 14 years since diagnosis and NSD‐ISS stages, female PwP had higher rates of falls, injuries, and fall‐related healthcare use. Frequent fallers had more severe motor and non‐motor symptoms compared to rare or never fallers. Notably, female sex emerged as a predictor of falls and related morbidity, despite a milder overall clinical phenotype.

Falls occurred in over one‐fourth of visits among PwP and increased with years since diagnosis and advancing disability, as indicated by NSD‐ISS stage 4 or higher. Importantly, female PwP had higher fall occurrence almost every year of follow‐up. By 7 years post‐diagnosis, two out of five female PwP reported a fall, increasing to almost nine out of ten by 14 years. Similar patterns were observed for injury and healthcare utilization, suggesting increasing severity with disease duration. Notably, approximately 15% of male and female PwP had already experienced a fall at the time of diagnosis. Although the injury rate at diagnosis was relatively low, this finding supports the need for fall screening from the time of diagnosis, as recommended in the American Academy of Neurology's universal neurology quality measurement set.^22^ Early screening can inform primary prevention strategies, such as physical therapy and home safety evaluations, and secondary prevention efforts, including assistive devices and bone health management.

Our findings should be interpreted in the context of the existing literature on falls in PD. In 22 prior studies, an average of 60.5% of PwP reported at least one fall.^23^ Among community‐dwelling PwP without dementia, similar to our study population, yearly fall rates have ranged from 21% to 65% when followed for up to 12 months.^24^, ^25^, ^26^, ^27^, ^28^, ^29^, ^30^, ^31^ Longitudinal studies have also described high rates of frequent falls. For example, in the Norwegian ParkWest study,^32^ 46% of newly diagnosed PwP experienced frequent falls by a median follow‐up time of 6 years. These rates are higher than those observed in our study, which may reflect length‐of‐time bias. Individuals with slower disease progression are more likely to remain in long‐term follow‐up, and thus overrepresented in our sample, potentially leading to an underestimation of the true frequency of falls. The relationship between falls and NSD‐ISS stage should be interpreted with caution. Although falls are not explicitly included in NSD‐ISS criteria, our fall classification was derived from MDS‐UPDRS Part II items, which also contribute to stages 3–6. This structural overlap may partially account for the observed association between higher stage and greater fall frequency. A sensitivity analysis excluding fall‐related staging components was not feasible due to lack of item‐level NSD‐ISS data in the public PPMI release. In addition, stage 6 was sparsely represented in our dataset (nine visits from five participants, all male), precluding assessment of sex differences. Nonetheless, fall rates increased progressively across stages, and the transition from NSD‐ISS stage 3–4 appeared to coincide with a marked rise in falls, suggesting a potential inflection point aligned with worsening functional impairment. This interpretation should be considered in the context that years since diagnosis and NSD‐ISS capture distinct constructs, with the former reflecting continuous time and the latter representing milestone‐based disease progression. Prospective studies incorporating independent and objective fall measures will be necessary to determine whether NSD‐ISS stage transitions independently predict fall risk beyond shared scale components.^33^

We also observed that frequent fallers had longer years since diagnosis, higher NSD‐ISS stages, greater functional impairment, and more severe motor and non‐motor symptoms compared to rare or never fallers. They showed higher H&Y stages, PIGD scores, and MDS‐UPDRS total and subscale scores, including greater motor complications on Part IV. In contrast, LEDD did not differ significantly across groups, suggesting dopaminergic dose alone may not explain fall risk. Instead, treatment‐related complications, rather than absolute dose, may contribute to greater fall vulnerability in frequent fallers. Cognitive decrements were most evident in processing speed, executive function, and visuospatial perception, with other domains showing no differences, underscoring the selective nature of cognitive vulnerability in PwP.^11^ OH rates trended higher with fall frequency but were confounded by age and disease duration, whereas SCOPA cardiovascular scores were significantly worse in frequent fallers, indicating patient‐reported OH symptoms may better capture fall risk than objective measures. This mismatch between objective and subjective OH symptoms and fall risk may be explained by differences in immediate versus delayed or symptomatic versus asymptomatic OH,^34^ which may contribute differently to fall risk.

Sex was a consistent predictor of fall risk and outcomes. Female PwP, despite lower LEDD needs, milder motor symptoms, and better cognitive performance, were significantly more likely than males to report falls, injuries, and healthcare use, even after adjustment for age, disease duration, and fall frequency. This excess risk was most evident for rare falls and upper extremity fractures; hip fractures did not reach significance due to low counts. Ongoing trials of osteoporosis treatment, a condition disproportionately affecting women, may help mitigate fracture risk in PD.^35^ Several factors may underlie sex differences. Biomechanically, females’ lower center of mass may enhance static stability but impair recovery from imbalance, while males’ higher center of mass may predispose to imbalance yet favor corrective movements.^36^ Female PwP also show higher fall risk independent of age,^37^, ^38^ despite fewer axial symptoms than males,^39^ and may be additionally influenced by urinary incontinence,^40^ traditional gendered expectations (eg, domestic tasks such as cooking and cleaning),^41^ a lower socioeconomic status,^42^ or a greater willingness to report falls and seek healthcare than their male counterparts.^43^ These findings highlight a disconnect between conventional severity markers and real‐world vulnerability in women with PD, underscoring the need for sex‐specific interventions.

Our study has several limitations. Differential withdrawal of participants with greater disability, including falls, may have led to underestimates in advanced PD. We did not evaluate the effect of physical activity, socioeconomic status, comorbidities, osteoporosis, or polypharmacy, all recognized fall risk factors, which may affect fall risk differently by sex. Fall definitions relied on self‐report from annual questionnaires rather than fall diaries (ie, the gold standard), raising the possibility of underreporting due to recall or social desirability bias (ie, underreporting to appear healthier). To reduce misclassification, we required consecutive years of fall reporting in our cross‐sectional analysis. Although our data span over 14 years since diagnosis, the mean duration for unique participants was 5 years, reflecting the later introduction of the fall questionnaire. Earlier enrollees generally had longer disease duration, while later enrollees had shorter, which we addressed by adjusting for years since diagnosis. Despite these limitations, the large multicenter cohort with multi‐year assessments and the ability to examine both disease‐duration and NSD‐ISS frameworks support the robustness of the findings.

## Conclusion

Our findings highlight the high burden of falls and fall‐related outcomes in PD, driven by disease progression and amplified by sex‐specific vulnerabilities. Fall risk reflected sex‐specific motor, cognitive, behavioral, and autonomic dysfunction, supporting a multifactorial model of susceptibility. These results highlight the importance of early screening, tailored prevention strategies, and attention to sex‐specific risks. Fall frequency may serve as a practical marker of disease progression and a target for sex‐specific interventions to reduce morbidity in PD.

## Author Roles

(1) Research Project: A. Conception, B. Organization, C. Execution; (2) Statistical Analysis: A. Design, B. Execution, C. Review and Critique; (3) Manuscript Preparation: A. Writing of the First Draft, B. Review and Critique.

J.A.V.: 1A, 1B, 1C, 2A, 2B, 3A.

K.H.: 1B, 1C, 2A, 2B, 3A.

D.E.L.: 3B.

M.T.D.: 3B.

A.E.: 3B.

B.L.: 3B.

D.S.B.: 2C, 3B.

A.S.: 2C, 3B.

## Disclosures

**Ethical Compliance Statement:** The study was approved by the institutional review boards at all participating PPMI sites and was conducted in accordance with applicable ethical standards and regulatory requirements. All participants provided written informed consent prior to enrollment, and consent was obtained and documented in accordance with institutional policies. We confirm that we have read the Journal's position on issues involved in ethical publication and affirm that this work is consistent with those guidelines.

**Funding Sources and Conflict of Interest:** JAV is an employee of the University of Pennsylvania and received funding support from the National Institutes of Health (1T32NS091006‐10), the Institute for Translational Medicine and Therapeutics of the Perelman School of Medicine at the University of Pennsylvania, and the Michael J. Fox Foundation; honoraria from the International Parkinson and Movement Disorder Society; and consultancy fees from the University City Science Center. KH is a graduate student at the University of Pennsylvania and received funding from the National Institutes of Health (5T32NS091006‐10). DEL is an employee of the University of Iowa. MTD is an employee of the University of Pennsylvania and received funding support from the National Institutes of Health (F30‐AG074524). AE is an employee of the University of Pennsylvania and received funding support from the National Institutes of Health and The Patient‐Centered Outcomes Research Institute.

BL is an employee of the University of Pennsylvania and received funding support from NINDS (DP1‐NS‐122038‐01), The Small Lake Foundation, Jonathan and Bonnie Rothberg, Neil and Barbara Smit. DSB is a faculty member at the University of Pennsylvania. AS is a faculty member at the University of Pennsylvania; consultancy fees in the past year from Acadia, Eli Lilly and Co, Neurocrine, Theravance, Cerevance, Spark/Roche, Boerhinger‐Ingelheim, Wave Life Sciences, Inhibikase, Prevail, Mitzubishi and Alertity Therapeutics. He has served on DSMBs for the Huntington Study Group and The Healey ALS Consortium (Massachusetts General Hospital). He has received grant funding from the Michael J. Fox Foundation, NIA and NINDS.

**Financial Disclosures for the Previous 12 Months:** The authors declare that there are no additional disclosures to report.

## PPMI STUDY TEAMS/CORES/COLLABORATORS FOR PUBLICATIONS

Executive Steering Committee:

Kenneth Marek, MD1 (Principal Investigator); Caroline Tanner, MD, PhD9; Tanya Simuni, MD3; Andrew Siderowf, MD, MSCE12; Douglas Galasko, MD27; Lana Chahine, MD41; Christopher Coffey, PhD4; Kalpana Merchant, PhD61; Kathleen Poston, MD40; Roseanne Dobkin, PhD43; Tatiana Foroud, PhD15; Brit Mollenhauer, MD8; Dan Weintraub, MD12; Ethan Brown, MD9; Karl Kieburtz, MD, MPH23; Mark Frasier, PhD6; Todd Sherer, PhD6; Sohini Chowdhury, MA6; Roy Alcalay, MD36 and Aleksandar Videnovic, MD47.

Steering Committee:

Duygu Tosun‐Turgut, PhD9; Werner Poewe, MD7; Susan Bressman, MD14; Jan Hammer15; Raymond James, RN22; Ekemini Riley, PhD42; John Seibyl, MD1; Leslie Shaw, PhD12; David Standaert, MD, PhD18; Sneha Mantri, MD, MS62; Nabila Dahodwala, MD12; Michael Schwarzschild47; Connie Marras45; Hubert Fernandez, MD25; Ira Shoulson, MD23; Helen Rowbotham2; Paola Casalin11, and Claudia Trenkwalder, MD8.

Michael J. Fox Foundation (Sponsor):

Todd Sherer, PhD; Sohini Chowdhury, MA; Mark Frasier, PhD; Jamie Eberling, PhD; Katie Kopil, PhD; Alyssa O’Grady; Maggie McGuire Kuhl; Leslie Kirsch, EdD and Tawny Willson, MBS.

Study Cores, Committees and Related Studies:

Project Management Core:

Emily Flagg, BA1.

Site Management Core:

Tanya Simuni, MD3; Bridget McMahon, BS1.

Strategy and technical operations:

Craig Stanley, PhD1; Kim Fabrizio, BA1.

Data Management Core:

Dixie Ecklund, MBA, MSN4; Trevis Huff, BSE4.

Screening Core:

Tatiana Foroud, PhD15; Laura Heathers, BA15; Christopher Hobbick, BSCE15; Gena Antonopoulos, BSN15.

Imaging Core:

John Seibyl, MD1; Kathleen Poston, MD40.

Statistics Core:

Christopher Coffey, PhD4; Chelsea Caspell‐Garcia, MS4; Michael Brumm, MS4.

Bioinformatics Core: Arthur Toga, PhD10; Karen Crawford, MLIS10.

Biorepository Core:

Tatiana Foroud, PhD15; Jan Hamer, BS15.

Biologics Review Committee:

Brit Mollenhauer8; Doug Galasko27; Kalpana Merchant61.

Genetics Core:

Andrew Singleton, PhD13.

Pathology Core:

Tatiana Foroud, PhD15; Thomas Montine, MD, PhD40.

Found:

Caroline Tanner, MD, PhD9.

PPMI Online:

Carlie Tanner, MD, PhD9; Ethan Brown, MD9; Lana Chahine, MD41; Roseann Dobkin, PhD43; Monica Korell, MPH9.

Site Investigators:

Charles Adler, PhD51; Roy Alcalay, MD36; Amy Amara, PhD52; Paolo Barone, PhD30; Bastiaan Bloem, PhD60; Susan Bressman, MD14; Kathrin Brockmann, MD26; Norbert Brüggemann, MD59; Lana Chahine, MD41; Kelvin Chou, MD44; Nabila Dahodwala, MD12; Alberto Espay, MD32; Stewart Factor, DO16; Hubert Fernandez, MD25; Michelle Fullard, MD52; Douglas Galasko, MD27; Robert Hauser, MD19; Penelope Hogarth, MD17; Shu‐Ching Hu, PhD21; Michele Hu, PhD58; Stuart Isaacson, MD31; Christine Klein, MD59; Rejko Krueger, MD2; Mark Lew, MD49; Zoltan Mari, MD56; Connie Marras, PhD45; Maria Jose Martí, PhD34; Nikolaus McFarland, PhD54; Tiago Mestre, PhD46; Brit Mollenhauer, MD8; Emile Moukheiber, MD28; Alastair Noyce, PhD63; Wolfgang Oertel, PhD64; Njideka Okubadejo, MD65; Sarah O’Shea, MD39; Rajesh Pahwa, MD48; Nicola Pavese, PhD57; Werner Poewe, MD7; Ron Postuma, MD55; Giulietta Riboldi, MD53; Lauren Ruffrage, MS18; Javier Ruiz Martinez, PhD35; David Russell, PhD1; Marie H. Saint‐Hilaire, MD22; Neil Santos, BS51; Wesley Schlett47; Ruth Schneider, MD23; Holly Shill, MD50; David Shprecher, DO24; Tanya Simuni, MD3; David Standaert, PhD18; Leonidas Stefanis, PhD38; Yen Tai, PhD29; Caroline Tanner, PhD9; Arjun Tarakad, MD20; Eduardo Tolosa, PhD34 and Aleksandar Videnovic, MD47.

Coordinators:

Susan Ainscough, BA30; Courtney Blair, MA18; Erica Botting19; Isabella Chung, BS56; Kelly Clark24; Ioana Croitoru35; Kelly DeLano, MS32; Iris Egner, PhD7; Fahrial Esha, BS53; May Eshel36; Frank Ferrari, BS44; Victoria Kate Foster57; Alicia Garrido, MD34; Madita. Grümmer59; Bethzaida Herrera50; Ella Hilt26; Chloe Huntzinger, BA52; Raymond James, BS22; Farah Kausar, PhD9; Christos Koros, MD, PhD38; Yara Krasowski60; Dustin Le, BS17; Ying Liu, MD52; Taina M. Marques, PhD2; Helen Mejia Santana, MA39; Sherri Mosovsky, MPH41; Jennifer Mule, BS25; Philip Ng, BS45; Lauren O’Brien; Abiola Ogunleye, PGDip29; Oluwadamilola Ojo, MD65; Obi Onyinanya, BS28; Lisbeth Pennente, BA31; Romina Perrotti55; Michael Pileggi, MS55; Ashwini Ramachandran, MSc12; Deborah Raymond, MS14; Jamil Razzaque, MS58; Shawna Reddie, BA46; Kori Ribb, BSN28; Kyle Rizer, BA54; Janelle Rodriguez, BS27; Stephanie Roman, HS1; Clarissa Sanchez, MPH20; Cristina Simonet, PhD29; Anisha Singh, BS23; Elisabeth Sittig 64; Barbara Sommerfeld MSN16;

Angela Stovall, BS44; Bobbie Stubbeman, BS32; Alejandra Valenzuela, BS49; Catherine Wandell, BS21; Diana Willeke8; Karen Williams, BA3 and Dilinuer Wubuli, MB45.

v. 15DEC2023Partners Scientific Advisory Board (Acknowledgement).

Funding: PPMI—a public‐private partnership—is funded by the Michael J. Fox Foundation for Parkinson's Research and funding partners, including 4D Pharma, Abbvie, AcureX, Allergan, Amathus Therapeutics, Aligning Science Across Parkinson's, AskBio, Avid Radiopharmaceuticals, BIAL, Biogen, Biohaven, BioLegend, BlueRock Therapeutics, Bristol‐Myers Squibb, Calico. Labs, Celgene, Cerevel Therapeutics, Coave Therapeutics, DaCapo Brainscience, Denali, Edmond J. Safra Foundation, Eli Lilly, Gain Therapeutics, GE HealthCare, Genentech, GSK, Golub Capital, Handl Therapeutics, Insitro, Janssen Neuroscience, Lundbeck, Merck, Meso Scale Discovery, Mission Therapeutics, Neurocrine Biosciences, Pfizer, Piramal, Prevail Therapeutics, Roche, Sanofi, Servier, Sun Pharma Advanced Research Company, Takeda, Teva, UCB, Vanqua Bio, Verily, Voyager. Therapeutics, the Weston Family Foundation and Yumanity Therapeutics.

1 Institute for Neurodegenerative Disorders, New Haven, CT.

2 University of Luxembourg, Luxembourg.

3 Northwestern University, Chicago, IL.

4 University of Iowa, Iowa City, IA.

5 VectivBio AG.

6 The Michael J. Fox Foundation for Parkinson's Research, New York, NY.

7 Innsbruck Medical University, Innsbruck, Austria.

8 Paracelsus‐Elena Klinik, Kassel, Germany.

9 University of California, San Francisco, CA.

10 Laboratory of Neuroimaging (LONI), University of Southern California.

11 BioRep, Milan, Italy.

12 University of Pennsylvania, Philadelphia, PA.

13 National Institute on Aging, NIH, Bethesda, MD.

14 Mount Sinai Beth Israel, New York, NY.

15 Indiana University, Indianapolis, IN.

16 Emory University of Medicine, Atlanta, GA.

17 Oregon Health and Science University, Portland, OR.

18 University of Alabama at Birmingham, Birmingham, AL.

19 University of South Florida, Tampa, FL.

20 Baylor College of Medicine, Houston, TX.

21 University of Washington, Seattle, WA.

22 Boston University, Boston, MA.

23 University of Rochester, Rochester, NY.

24 Banner Research Institute, Sun City, AZ.

25 Cleveland Clinic, Cleveland, OH.

26 University of Tübingen, Tübingen, Germany.

27 University of California, San Diego, CA.

28 Johns Hopkins University, Baltimore, MD.

29 Imperial College of London, London, UK.

30 University of Salerno, Salerno, Italy.

31 Parkinson's Disease and Movement Disorders Center, Boca Raton, FL.

32 University of Cincinnati, Cincinnati, OH.

34 Hospital Clinic of Barcelona, Barcelona, Spain.

35 Hospital Universitario Donostia, San Sebastian, Spain.

36 Tel Aviv Sourasky Medical Center, Tel Aviv, Israel.

37 St. Olav's University Hospital, Trondheim, Norway.

38 National and Kapodistrian University of Athens, Athens, Greece.

39 Columbia University Irving Medical Center, New York, NY.

40 Stanford University, Stanford, CA.

41 University of Pittsburgh, Pittsburgh, PA.

42 Center for Strategy Philanthropy at Milken Institute, Washington D.C.

43 12, New Brunswick, NJ.

44 University of Michigan, Ann Arbor, MI.

45 Toronto Western Hospital, Toronto, Canada.

46 The Ottawa Hospital, Ottawa, Canada.

47 Massachusetts General Hospital, Boston, MA.

48 University of Kansas Medical Center, Kansas City, KS.

49 University of Southern California, Los Angeles, CA.

50 Barrow Neurological Institute, Phoenix, AZ.

v. 15DEC202351 Mayo Clinic Arizona, Scottsdale, AZ.

52 University of Colorado, Aurora, CO.

53 NYU Langone Medical Center, New York, NY.

54 University of Florida, Gainesville, FL.

55 Montreal Neurological Institute and Hospital/McGill, Montreal, QC, Canada.

56 Cleveland Clinic‐Las Vegas Lou Ruvo Center for Brain Health, Las Vegas, NV.

57 Clinical Aging Research Unit, Newcastle, UK.

58 John Radcliffe Hospital Oxford and Oxford University, Oxford, UK.

59 Universität Lübeck, Luebeck, Germany.

60 Radboud University, Nijmegen, Netherlands.

61 TransThera Consulting.

62 Duke University, Durham, NC.

63 Wolfson Institute of Population Health, Queen Mary University of London, UK.

64 Philipps‐University Marburg, Germany.

65 University of Lagos, Nigeria.

v. 15DEC2023.

## Financial Disclosures and Conflicts of Interest

Author disclosures are available in the Supporting Information.

## Supporting information


**Table S1.** Fall frequency classification based on unified Parkinson's disease rating scale items 13 and 14
**Table S2.** Frequency of falls, injury, and healthcare utilization between PD, PAS, and HC
**Table S3.** Effect of cohort between PD and PAS on injury and healthcare utilization
**Table S4.** Effect of fall frequency and sex on injury and healthcare utilization measures in fallers with PD and PAS
**Table S5.** Falls, injury, and healthcare utilization across NSD‐ISS stages in PD patients
**Figure S1.** Sampling without replacement for cross‐sectional analysis.
**Figure S2.** Motor differences between never, rare, and frequent fallers in PD.
**Figure S3.** Non‐motor differences between never, rare, and frequent fallers in PD.
**Figure S4.** Sex‐based differences in the cross‐sectional PD cohort.
**Figure S5.** Outcome differences between rare and frequent fallers in PD.

**Data S1.** Coi_disclosure.

## Data Availability

Data used in the preparation of this article were obtained on December 11, 2024 and October 1, 2025 from the Parkinson's Progression Markers Initiative (PPMI) database (https://www.ppmi-info.org/access-data-specimens/download-data), RRID:SCR_006431. For up‐to‐date information on the study, visit http://www.ppmi-info.org. This analysis used data openly available from the PPMI (Tier 1 data). https://github.com/KHefter/PPMIFallsAndInjury.
