# Supplementary material for: Sex Differences in Fall Frequency, Risk Factors, and Outcomes in Parkinson's Disease: A Cross‐Sectional Analysis
Source: Mov Disord Clin Pract. 2026 May 7;13(10):2458–73. doi: 10.1002/mdc3.70664 (PMC13339450; doi:10.1002/mdc3.70664)
Supplement: Supplementary file 1 [file MDC3-13-2458-s001.docx]

**Supplementary Material**

**Sex Differences in Fall Frequency, Risk Factors, and Outcomes in Parkinson’s Disease: A Cross-Sectional Analysis**

Joaquin A. Vizcarra, MD*;^1,2^ Kat Hefter, JD, MSE*;^2,3^ David-Erick Lafontant MS;^4^ Michael Tran Duong, MD, PhD;^5^ Ashkan Ertefaie, PhD;^6^ Brian Litt, MD;^1,2^ Dani S. Bassett, PhD;^3^ Andrew Siderowf, MD, MSCE^1^ and The Parkinson’s Progression Markers Initiative

1. Department of Neurology, University of Pennsylvania Perelman School of Medicine, Philadelphia, PA, USA
2. Center for Neuroengineering and Therapeutics, University of Pennsylvania, Philadelphia, PA 19104, USA
3. Department of Bioengineering, University of Pennsylvania School of Engineering and Applied Sciences, Philadelphia, PA, USA
4. Department of Biostatistics, College of Public Health, University of Iowa, Iowa City, IA, USA
5. Department of Radiology, University of Pennsylvania Perelman School of Medicine, Philadelphia, PA, USA
6. Department of Biostatistics, Epidemiology & Informatics, University of Pennsylvania, Philadelphia, PA, USA

*These two authors contributed equally to this work

**Contents:**

**eTable 1: Fall Frequency Classification Based on Unified Parkinson’s Disease Rating Scale Items 13 and 14**

**eTable 2: Frequency of Falls, Injury, and Healthcare Utilization Between PD, PAS, and HC**

**eTable 3: Effect of Cohort Between PD and PAS on Injury and Healthcare Utilization**

**eTable 4: Effect of Fall Frequency and Sex on Injury and Healthcare Utilization Measures in Fallers with PD and PAS**

**eTable 5: Falls, Injury, and Healthcare Utilization across NSD-ISS Stages in PD Patients**

**eFigure 1: Sampling without Replacement for Cross-sectional Analysis**

**eFigure 2: Motor Differences between Never, Rare, And Frequent Fallers in PD**

**eFigure 3: Non-Motor Differences Between Never, Rare, and Frequent Fallers in PD**

**eFigure 4: Sex-based Differences in the Cross-Sectional PD Cohort**

**eFigure 5: Outcome Differences between Rare and Frequent Fallers in PD**

**eTable 1: Fall Frequency Classification Based on Unified Parkinson’s Disease Rating Scale Items 13 and 14**

| *Fall frequency* | *Item 13 score* | *Combination* | *Item 14 score* |
| --- | --- | --- | --- |
| *None* | 0 = none | AND | ≤2 (0 = none; 1 = rare freezing when walking; 2 = occasional freezing when walking) |
| *Rare* | 1 = rare falling | AND | ≤2 (0 = none; 1 = rare freezing when walking; 2 = occasional freezing when walking) |
| *Frequent* | ≥2 (2 = occasionally falls; 3 = falls an average of once daily; 4 = falls more than once daily) | OR | ≥3 (3 = occasionally falls from freezing; 4 = frequent falls from freezing) |

Fall frequency was determined by the following response combinations on Unified Parkinson’s Disease Rating Scale items 13 and 14.

**eTable 2: Frequency of Falls, Injury, and Healthcare Utilization Between PD, PAS, and HC**

|  | **PD**  **(n = 2374 visits)** | **PAS**  **(n = 3848 visits)** | | | **HC**  **(n = 757 visits)** | | |
| --- | --- | --- | --- | --- | --- | --- | --- |
|  | **N (%)** | **N (%)** | **OR**  **(95% CI)** | **p** | **N (%)** | **OR**  **(95% CI)** | **p** |
| **Falls** | | | | | | | |
| Any Falls | 677 (28.5%) | 850 (22.1%) | 1.66 *  (1.46, 1.87) | <0.001 | 80 (10.6%) | 4.03 *  (3.14, 5.23) | <0.001 |
| Rare Falls | 514 (21.7%) | 806 (20.9%) | 1.22 *  (1.06, 1.38) | 0.003 | 78 (10.3%) | 2.75 *  (2.14, 3.59) | <0.001 |
| Frequent Falls | 163 (6.9%) | 44 (1.1%) | 6.49 *  (4.66, 9.24) | <0.001 | 2 (0.3%) | 33.2 *  (10.5, 201.1) | <0.001 |
| **Injuries** | | | | | | | |
| Any Injury | 264 (11.1%) | 303 (7.9%) | 1.70 *  (1.42, 2.04) | <0.001 | 33 (4.4%) | 3.26 *  (2.27, 4.85) | <0.001 |
| Multiple Injuries | 70 (2.9%) | 65 (1.7%) | 1.85 *  (1.31, 2.64) | <0.001 | 5 (0.7%) | 6.32 *  (2.27, 18.2) | 0.001 |
| Any Fracture | 80 (3.4%) | 96 (2.5%) | 1.55 *  (1.14, 2.11) | 0.005 | 10 (1.3%) | 3.36 *  (1.80, 7.01) | 0.005 |
| Hip Fracture | 18 (0.8%) | 32 (0.8%) |  | NS | 2 (0.3%) |  | NS |
| Upper Extremity Fracture | 32 (1.3%) | 31 (0.8%) | 2.03 *  (1.23, 3.38) | 0.006 | 6 (0.8%) | 2.14  (0.94, 5.74) | 0.09 |
| Skull Fracture | 4 (0.2%) | 2 (0.1%) |  | NS | 0 (0%) |  | NS |
| Other Fracture | 35 (1.5%) | 37 (1.0%) | 1.61  (1.00, 2.56) | 0.049 | 2 (0.3%) | 7.00 *  (2.11, 43.3) | 0.008 |
| Head Injury | 85 (3.6%) | 82 (2.1%) | 1.78 *  (1.29, 2.42) | <0.001 | 8 (1.1%) | 4.60 *  (2.33, 10.5) | <0.001 |
| Lacerations | 48 (2.0%) | 20 (0.5%) | 3.92 *  (2.34, 6.82) | <0.001 | 5 (0.7%) | 4.03 *  (1.73, 11.7) | 0.004 |
| Other Injury | 139 (5.9%) | 177 (4.6%) | 1.50 *  (1.19, 1.89) | <0.001 | 18 (2.4%) | 2.85 *  (1.77, 4.86) | <0.001 |
| **Healthcare Utilization** | | | | | | | |
| Any HC Use | 202 (8.5%) | 226 (5.9%) | 1.71 *  (1.39, 2.09) | <0.001 | 23 (3.0%) | 3.81 *  (2.48, 6.12) | <0.001 |
| Outpatient Visit | 112 (4.7%) | 149 (3.9%) | 1.39 *  (1.07, 1.79) | 0.012 | 13 (1.7%) | 3.61 *  (2.08, 6.82) | <0.001 |
| ED Visit | 138 (5.8%) | 130 (3.4%) | 1.99 *  (1.54, 2.56) | <0.001 | 15 (2.0%) | 3.97 *  (2.37, 7.16) | <0.001 |
| Hospitalization | 40 (1.7%) | 27 (0.7%) | 2.46 *  (1.50, 4.10) | <0.001 | 7 (0.9%) | 2.54  (1.19, 6.33) | 0.026 |
| Surgery | 29 (1.2%) | 34 (0.9%) |  | NS | 8 (1.1%) |  | NS |
| Institutionalization | 5 (0.2%) | 0 (0%) |  | NS | 1 (0.1%) |  | NS |

ED, Emergency Department; HC, Healthy Control; NS, not significant on the Wald test; OR, Odds ratio; PD, Parkinson’s disease; PAS, Prodromal alpha-synucleinopathy. *: statistically significant from PD, p<0.028 (BH threshold for FDR 0.05). Model also conditioned on age and sex (odds ratios/significance not shown). OR calculated as PD/another cohort. OR only calculated for variables with models for which cohort membership provided a significant improvement (p<0.05 using Wald test) over models without cohort membership.

**eTable 3: Effect of Cohort Between PD and PAS on Injury and Healthcare Utilization**

|  | **PD**  **(n = 514 rare fall visits**  **n = 163 frequent fall visits)** | **PAS**  **(n = 806 rare fall visits**  **n = 44 frequent fall visits)** |  | |
| --- | --- | --- | --- | --- |
|  | ***N (%)*** | ***N (%)*** | ***OR (95% CI)*** | ***p*** |
| Any Injury | 264 (39.0%) | 303 (35.6%) | 1.16 (0.89, 1.40) | 0.35 |
| Fracture: | 80 (11.8%) | 96 (11.3%) | 1.01 (0.71, 1.42) | 0.97 |
| Hip Fracture | 18 (2.7%) | 32 (3.8%) | 0.75 (0.39, 1.39) | 0.36 |
| Upper Extremity Fracture | 32 (4.7%) | 31 (3.6%) | 1.26 (0.72, 2.18) | 0.41 |
| Skull Fracture | 4 (0.6%) | 2 (0.2%) | 2.50 (0.44, 19.14) | 0.31 |
| Other Fracture | 35 (5.2%) | 37 (4.4%) | 1.08 (0.65, 1.80) | 0.76 |
| Head Injury | 85 (12.6%) | 82 (9.6%) | 1.14 (0.80, 1.62) | 0.48 |
| Laceration | 48 (7.1%) | 20 (2.4%) | 2.17 * (1.23, 3.94) | 0.008 |
| Other Injury | 139 (20.5%) | 177 (20.8%) | 0.96 (0.73, 1.25) | 0.76 |
| Multiple Injuries | 70 (10.3%) | 65 (7.64%) | 1.07 (0.72, 1.58) | 0.75 |
| Any HC Use | 202 (29.8%) | 226 (26.6%) | 1.07 (0.83, 1.36) | 0.61 |
| Doctor Visit | 112 (16.5%) | 149 (17.5%) | 0.83 (0.62, 1.11) | 0.21 |
| ED Visit | 138 (20.4%) | 130 (15.3%) | 1.22 (0.92, 1.65) | 0.17 |
| Hospitalization | 40 (5.9%) | 27 (3.2%) | 1.59 (0.92, 2.75) | 0.09 |
| Surgery | 29 (4.3%) | 34 (4.0%) | 0.96 (0.54, 1.66) | 0.88 |
| Institutionalization | 5 (0.7%) | 0 (0%) | n/a | n/a |

Differences in injury and healthcare utilization measures for fallers with PD and PAS. OR, odds ratio: PD/PAS. Parkinson’s disease; PAS, Prodromal alpha-synucleinopathy. ORs calculated from a model conditioned on age, fall frequency, sex, and cohort. Results for OR based on fall frequency and sex are in Supplementary Table 4. *: statistically significant difference between PD / PAS, p<0.024 (BH threshold for FDR 0.05). m.p. = machine precision.

**eTable 4: Effect of Fall Frequency and Sex on Injury and Healthcare Utilization Measures in Fallers with PD and PAS**

|  | **Fall Frequency** | | | | **Sex** | | | |
| --- | --- | --- | --- | --- | --- | --- | --- | --- |
|  | ***Rare Fallers***  ***N (%)*** | ***Frequent Fallers***  ***N (%)*** | ***OR***  ***(95% CI)*** | ***p*** | ***Male***  ***N (%)*** | ***Female N (%)*** | ***OR***  ***(95% CI)*** | ***p*** |
| Total visits | 1320 | 207 |  |  | 727 | 800 |  |  |
| **Injuries** | | | | | | | | |
| Any Injury | 461 (34.9%) | 106 (51.2%) | 1.95 *  (1.42, 2.67) | <0.001 | 248 (34.1%) | 319 (39.9%) | 1.50 *  (1.20, 1.88) | <0.001 |
| Multiple Injuries | 94 (7.1%) | 41 (19.8%) | 2.88 *  (1,84, 4.44) | <0.001 | 68 (9.4%) | 67 (8.4%) | 1.27  (0.86, 1.87) | 0.22 |
| Any Fracture | 140 (10.6%) | 36 (17.4%) | 1.74 *  (1.12, 2.66) | 0.014 | 74 (10.2%) | 102 (12.8%) | 1.62 *  (1.15, 2.29) | 0.006 |
| Hip Fracture | 42 (3.2%) | 8 (3.9%) | 1.42  (0.58, 3.09) | 0.40 | 16 (2.2%) | 34 (4.3%) | 2.30 *  (1.23, 4.49) | 0.01 |
| Upper Extremity Fracture | 45 (3.4%) | 18 (8.7%) | 2.77 *  (1.47, 5.08) | 0.001 | 21 (2.9%) | 42 (5.3%) | 2.67 *  (1.51, 4.85) | 0.001 |
| Skull Fracture | 5 (0.4%) | 1 (0.5%) | 0.70  (0.03, 4.95) | 0.76 | 3 (0.4%) | 3 (0.4%) | 1.94  (0.32, 11.90) | 0.46 |
| Other Fracture | 59 (4.5%) | 13 (6.3%) | 1.23  (0.61, 2.31) | 0.54 | 39 (5.4%) | 33 (4.1%) | 0.88  (0.53, 1.46) | 0.62 |
| Head Injury | 129 (9.8%) | 38 (18.4%) | 1.74 *  (1.12, 2.64) | 0.01 | 89 (12.2%) | 78 (9.8%) | 1.00  (0.71, 1.42) | 0.98 |
| Laceration | 41 (3.1%) | 27 (13.0%) | 3.07 *  (1.76, 5.30) | <0.001 | 43 (5.9%) | 25 (3.1%) | 0.85  (0.49, 1.46) | 0.56 |
| Other Injury | 258 (19.5%) | 58 (28.0%) | 1.77*  (1.24, 2.51) | 0.02 | 130 (17.9%) | 186 (23.3%) | 1.46 *  (1.12, 1.91) | 0.005 |
| **HC Utilization** | | | | |  |  |  |  |
| Any HC Utilization | 336 (25.5%) | 92 (44.4%) | 2.27 *  (1.64, 3.14) | <0.001 | 193 (26.5%) | 235 (29.4%) | 1.48 *  (1.16, 1.89) | 0.002 |
| Outpatient Visit | 205 (15.5%) | 56 (27.1%) | 2.14 *  (1.47, 3.08) | <0.001 | 115 (15.8%) | 146 (18.3%) | 1.41 *  (1.06, 1.89) | 0.02 |
| ED Visit | 199 (15.1%) | 69 (33.3%) | 2.55 *  (1.79, 3.62) | <0.001 | 124 (17.1%) | 144 (18.0%) | 1.49 *  (1.11, 1.99) | 0.007 |
| Hospitalization | 49 (3.7%) | 18 (8.7%) | 1.75  (0.94, 3.17) | 0.07 | 37 (5.1%) | 30 (3.8%) | 1.11  (0.65, 1.90) | 0.68 |
| Surgery | 47 (3.6%) | 16 (7.7%) | 2.34 *  (1.21, 4.35) | 0.009 | 26 (3.6%) | 37 (4.6%) | 1.63  (0.95, 2.86) | 0.08 |
| Institutionalization | 4 (0.3%) | 1 (0.5%) | 0.76  (0.04, 5.38) | 0.82 | 3 (0.4%) | 2 (0.3%) | 1.07  (0.14, 6.78) | 0.94 |

HC, Healthcare; ED, Emergency Department; OR, Odds Ratio; Parkinson’s disease; PAS, Prodromal alpha-synucleinopathy. *: statistically significant difference between rare/frequent fallers or male/female, p<0.024 (BH threshold for FDR 0.05). m.p. = machine precision. Model conditioned on age (odds significance not shown). OR: frequent/rare; female/male.

**eTable 5: Falls, Injury, and Healthcare Utilization across NSD-ISS stages in PD patients**

| **NSD Stage** | **Total Visits** | **Fall Occurrences** | **Rare Falls** | **Frequent Falls** | **Any Injury** | **Any Healthcare Use** |
| --- | --- | --- | --- | --- | --- | --- |
| **2a** | 4 | 0 (0%) | 0 (0%) | 0 (0%) | 0 (0%) | 0 (0%) |
| **2b** | 258 | 22 (8.5%) | 22 (8.5%) | 0 (0%) | 8 (3.1%) | 5 (1.9%) |
| **3** | 1120 | 229 (20.4%) | 205 (18.3%) | 24 (2.1%) | 80 (7.1%) | 60 (5.4%) |
| **4** | 561 | 253 (45.1%) | 181 (32.3%) | 72 (12.8%) | 101 (18.0%) | 83 (14.8%) |
| **5** | 83 | 63 (75.9%) | 24 (28.9%) | 39 (47.0%) | 37 (44.6%) | 29 (34.9%) |
| **6** | 9 | 8 (88.8%) | 1 (11.1%) | 7 (77.8%) | 7 (77.8%) | 6 (66.7%) |

Values are counts with corresponding percentages.

**eFigure 1: Sampling without Replacement for Cross-sectional Analysis**

Of 937 unique PD patients in the PPMI, we conducted sampling without replacement for individuals with two consecutive years of available fall data. After sampling, 332 unique participants did not fulfill consecutive years’ definitions and were removed from the cross-sectional analysis.

**eFigure 2: Motor Differences between Never, Rare, And Frequent Fallers in PD**

Boxplot displays the distribution of values across three groups. The central line represents the median, boxes indicate the interquartile range (IQR), and whiskers extend to 1.5 × IQR. Outliers are shown as individual points. Group differences were assessed using linear regressions adjusted for age, sex, and years since diagnosis. H&Y, Hoehn and Yahr; PIGD, Postural Instability and Gait Disorder; MDS-UPDRS, Movement Disorder Society Unified Parkinson’s Disease Rating Scale; S&E, Modified Schwab and England Activities of Daily Living Scale. ON denotes the ON medication state. *: statistically significant from rare, p<0.016 (BH threshold for FDR 0.05).

**eFigure 3: Non-Motor Differences Between Never, Rare, and Frequent Fallers in PD**

Boxplot displays the distribution of values across three groups. The central line represents the median, boxes indicate the interquartile range (IQR), and whiskers extend to 1.5 × IQR. Outliers are shown as individual points. Group differences were assessed using linear regressions adjusted for age, sex, and years since diagnosis. MoCA, Montreal Cognitive Assessment; BJLO, Benton Judgment of Line Orientation; SDMT, Symbol Digit Modalities Test; TMT, Trail Making Test, GDS-15, Geriatric Depression Scale; STAI, State-Trait Anxiety Inventory; SCOPA, Scales for Outcomes in Parkinson’s Disease – Autonomic. *: statistically significant from rare, p<0.016 (BH threshold for FDR 0.05).

**eFigure 4: Sex-based Differences in the Cross-Sectional PD Cohort**

For continuous variables, boxplots display the distribution of values across two groups. The central line represents the median, boxes indicate the interquartile range (IQR), and whiskers extend to 1.5 × IQR. Outliers are shown as individual points. For categorical variables, bar plots show the percentage of each outcome in each group. Group differences were assessed using regressions adjusted for age, fall frequency, and years since diagnosis. Regressions were linear for continuous variables and logistic for categorical variables.

LEDD, Levodopa Equivalent Daily Dose; MDS-UPDRS, Movement Disorder Society Unified Parkinson’s Disease Rating Scale; BJLO, Benton Judgement of Line Orientation; HVLT, Hopkins Verbal Learning Test; LFLT, Lexical Fluency Letter Test; SDMT, Symbol Digit Modalities Test; TMT, Trail Making Test; ESS, Epworth Sleepiness Scale; RBQ, REM Behavioral Disorder; SCOPA, Scales for Outcomes in Parkinson’s Disease – Autonomic. *: statistically significant from rare, p<0.016 (BH threshold for FDR 0.05).

**eFigure 5: Outcome Differences between Rare and Frequent Fallers in PD**

Bar plot shows the percentage of each outcome in each group. Group differences were assessed using logistic regressions adjusted for age, sex, and years since diagnosis. Frequent fallers showed significant differences from rare fallers for injuries, lacerations, multiple injuries, and ED visits. *: statistically significant from rare, p<0.016 (BH threshold for FDR 0.05).
